# Analysis of Agricultural Land Use Change in the Middle Reach of the Heihe River Basin, Northwest China

**DOI:** 10.3390/ijerph110302698

**Published:** 2014-03-04

**Authors:** Li Fu, Lanhui Zhang, Chansheng He

**Affiliations:** 1 Key Laboratory of Digital Earth Science, Institute of Remote Sensing and Digital Earth, Chinese Academy of Sciences, Beijing 100101, China; E-Mail: fuli@irsa.ac.cn; 2 Department of Geography, Western Michigan University, Kalamazoo, MI 49008, USA; 3 Center for Dryland Water Resources Research and Watershed Sciences, Key Laboratory of Western China’s Environmental Systems (MOE), Lanzhou University, Lanzhou 730000, China; E-Mail: zhanglanh06@lzu.cn

**Keywords:** Heihe River Basin, land use and land cover change (LULCC), agriculture, discharge

## Abstract

The Heihe River Basin (HRB) is the second largest inland river basin in arid Northwest China. The expanding agricultural irrigation, growing industrialization, and increasing urban development in the middle reach have depleted much of the river flow to the lower reach, degrading the corresponding ecosystems. Since the enactment of the State Council of China’s new HRB water allocation policy in 2000 tremendous land use and land cover (LULC) changes have taken place to reduce water consumption in the middle reach and deliver more water downstream. This paper analyzes LULC changes during the period of 2000–2009 to understand how the changing land use patterns have altered water resource dynamics in the region. Results, while yet to be further verified in the field, show that from 2000 to 2009, urban, agricultural land, rangeland, and forest areas have increased, and barren area has decreased. Within the cropland, rice (a high water consumption crop) planting area decreased, while corn and wheat (relatively lower water consumption crops) planting areas increased. These changes in land use patterns, especially in the agricultural zones, have ensured the discharge of the required amount of water to the lower reach.

## 1. Introduction

Land use and land cover (LULC) changes, such as the conversion of land use types like grasslands, woodlands and forests into croplands and pastures are responsible for contributing 20%–75% of all atmospheric emissions of greenhouse gases [1], and for causing deteriorating ecological diversity, soil fertility, water and air quality [2,3,4]. Different approaches and datasets have been used to detect and understand LULC changes and the related driving forces. For example, Serra and colleagues [5] analyzed LULC changes in the Mediterranean region by using a hybrid classifier of remote sensing data and explored the main driving forces of the LULC using a multiple logistic regression method. The authors stated that the environmental protection polices and expansion of tourism led to the LULC change, and the increase of irrigated herbaceous crops within the region would lead to greater agricultural water demands and intensify conflicts among different water uses [5]. LULC detection is also important for crop irrigation and water resources management at the watershed scale [6]. A study in the Indus Basin using multi-temporal remote sensing-based products captured seasonal phenological information of different crops for crop classification, and subsequently used such information to identify different water users and to formulate water management plans [7]. Schilling *et al.* [8] analyzed the impacts of shifting crop pattern from mixed perennial/annual cropping system to an annual system of corn and soybean row crops over the last century in the corn belt region of the United States, and reported that the changing crop patterns have altered the water balance of the Raccoon River watershed, decreasing evapotranspiration (ET) and increasing streamflow and base flow. In a similar study, Schilling *et al.* [9] using statistical analysis of annual discharge and precipitation in the Mississippi River, indicate that expanding soybean acreage in the basin is the main factor that caused the increasing discharge of the Upper Mississippi River Basin. Moreover, LULC change affects not only the quantity of water but also the quality of water [10,11].

LULC changes in China, as elsewhere, are being driven by multiple demands for food production, industrial development, and urban expansion for the growing population [12]. Supported by a dynamic LULC information system, Liu and his colleagues [13,14] implemented China’s land use change detection program during the mid-1990s to 2000. The results revealed that a great portion of the arable land in China’s traditional agricultural zones had been occupied by built-up and residential areas, while changes in production conditions, economic benefits and climate conditions had led to the reclamation of arable land in northern China [13,14]. They also reported the initial success of the “returning arable land into woodland or grassland” policies [14]. Another study by Weng [15] investigated the land use change dynamics in the Zhujiang Delta by the combined use of remote sensing and geographic information system (GIS), and reported notably uneven urban growth and tremendous cropland losses in this fast growing coastal region. A similar research in this region conducted by Li [16] compared the differences between the western and eastern development corridors in the Zhujiang region, identified the spatial dependency of land use changes, and highlighted the influence of enforcing land use polices on the direction and magnitude of landscape changes. Another study by Xiao *et al.* [17] in Shijiazhuang City revealed that governmental policies were among the major drivers that influenced the LULC change in the city [17]. Many studies such as those discussed above have focused on the LULC change patterns in the fast growing metropolitan areas in eastern China, relatively few studies have addressed LULC change patterns in agricultural watersheds in arid Northwest China, particularly their impacts on water resources [12,18]. This research, through a case study of the Heihe River Basin (HRB), analyzes the pattern and rates of LULC changes in the middle reach and evaluates the impacts of these changes on streamflow discharges downstream for ecosystem protection.

**Figure 1:**
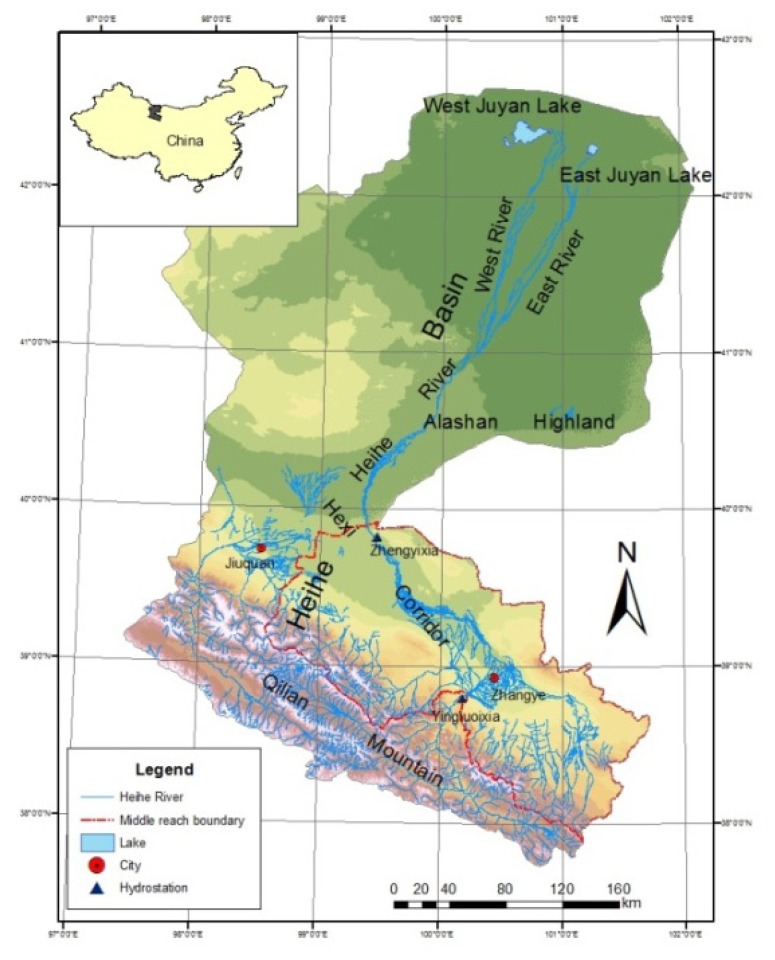
The boundary of the Heihe River Basin in Northwest China.

The HRB (Figure 1) is the second largest inland river basin in arid Northwest China. The middle reach of the HRB has an agricultural history going back over 2,000 years owning to the flat land, adequate sunlight, and convenient water sources coming from the Qilian Mountains [19]. 

Human activities like reclaiming fallow land, constructing water supply projects and developing irrigated agriculture, have changed the natural landscape significantly [18,19]. Over the past few decades, the increased withdrawals for agricultural irrigation and municipal water supplies in the Hexi Corridor of the HRB since the 1970s have depleted much of the river flows to the lower reach, shrinking the East Juyan Lake and drying up the West Juyan Lake, endangering aquatic ecosystems and accelerating desertification [20]. To mitigate the damages to the ecosystem and improve the living conditions in the lower reach, the State Council of China issued an executive order in 2000, mandating that Gansu Province distribute about 0.95 × 10^9^ m³ water per year under the normal climate years to the lower reach users. To implement the mandate, since 2000 local governments in the middle reach (particularly, Zhangye City) have made adjustments to the dominant crops and cropping patterns, most notably reducing or eliminating rice planting and increasing corn planting to save more water for downstream users [20]. Few studies have evaluated the LULC changes and their feedbacks to the water resources allocation in the HRB region. Thus, this study was undertaken to: (1) identify the LULC change patterns in the middle reach of the HRB between 2000 and 2009, especially in the cropland zones; and (2) assess the influences of LULC changes on water resource distributions in the study area. 

## 2. Methods

### 2.1. Study Area

The HRB, the second largest inland river basin in arid Northwest China, is located between 96°42′~102°00′E and 37°41′~ 42°42′N and covers an area of approximately 128,000 km² (Figure 1). Sandwiched between the southern Qilian Mountains and the northern Alashan Highlands, the middle reach of the HRB is like a corridor trending from northwest to southeast, with elevation ranges between 1,300 and 2,500 m [19,20]. The climate is characterized as arid, with mean annual precipitation varying from about 331 mm in the southern mountainous areas to less than 100 mm in the northern high-plain areas [19]. The inter-annual variability of the precipitation is as high as 80%, and over 60% of the precipitation falling between June and August [19]. The mean annual air temperature is 8 °C in the lower part (northern) of the basin and decreases to 2.1 °C in the south [19]. The most common vegetation in the area includes temperate dwarf shrub and subshrub desert vegetation dominated by *Chenopodiaceae*, *Zygophyllaceae*, *Ephedranceae*, *Asteraceae*, *Poaceae*, and *Leguminosae* species [19].

The Hexi Corridor, located in the middle reach of HRB, is an important source of commodity grain in China, supporting more than 97% of the HRB’s 1.8 million inhibits in two metropolitan areas: Zhangye (population 1.25 million in 2000) and Jiuquan (population 0.49 million in 2000) [20]. As the largest oasis in the middle reach, the Hexi Corridor is the major water consumer in the HRB, where 86% of the water withdrawal from the Heihe River was used to irrigate the farmland in the Corridor [20]. The main crops are spring wheat, corn and rice. Spring wheat is generally sown in late March and harvested during the middle of July, corn and rice are planted in April and harvested during September [20].

### 2.2. Data Sources

Two sets of 30 m resolution Landsat 5 and 7 images, acquired in 2000 and 2009, respectively, were downloaded from the United States Geological Survey (USGS) website (http://earthexplorer.usgs.gov/) [21] for mapping the 2000 and 2009 LULC types in the middle reach of the HRB (Table 1). The collected satellite images were taken in the growing season of the plants for better distinguishing vegetation from other land cover types. Additionally, ancillary data of 2000 LULC map of the HRB and 2008 air photo of Zhangye City were also collected to assist the classification of the LULC types. Agricultural statistics data, streamflow data from the Zhengyixia gauge station (outlet of the middle reach) and Yingluoxia gauge station (outlet of the upper reach), the 2000–2009 precipitation data of Qilian weather station were also acquired to analyze the influences of the LULC changes on water allocation in the study area. All of these data were obtained from the Cold and Arid Regions Environmental and Engineering Research Institute, Chinese Academy of Sciences.

**Table 1:** Satellite images used in this study.

Date	Path-Row	Satellite	Sensor	Cloud Cover	Preprocessing
10 August 2000	133-33	Landsat-7	ETM+	<10%	Geometric Correction
10 August 2000	133-34	Landsat-7	ETM+	<10%	Geometric Correction
14 June 2000	134-32	Landsat-7	ETM+	<10%	Geometric Correction
14 June 2000	134-33	Landsat-7	ETM+	<10%	Geometric Correction
8 November 2009	133-33	Landsat-5	TM	<10%	Geometric Correction
8 November 2009	133-34	Landsat-5	TM	<10%	Geometric Correction
17 July 2009	134-32	Landsat-5	TM	<10%	Geometric Correction
17 July 2009	134-33	Landsat-5	TM	<10%	Geometric Correction

### 2.3. Image Processing

The collected images have already been geo-referenced to WGS84 UTM Zone 47N. These images were then radiometrically corrected using the calibration utility for Landsat in ENVI 4.7^TM^ software package. Atmospheric correction was also conducted in ENVI, using the automatic calculated dark object method, to reduce the influence of atmospheric effects from the different images. The pre-processed images were subsequently clipped to the boundary of the study area. 

Supervised classification method was used to map the 2000 and 2009 land cover types. The Chinese Land Resource Classification System, from the Cold and Arid Regions Environmental and Engineering Research Institute of Chinese Academy of Sciences, was used as the classification scheme to categorize the pixels of the two images. The classification system includes 6 major types (Level 1), *i.e.*, cropland, forestland, grassland, water, urban and/or built-up land, and barren land. These types were then further discriminated into more detailed land cover types based on the differences in land characteristics, coverage and uses (Level 2, Table 2). Because the paved surfaces of urban area and barren land/or desert in the study area both have similar reflectance values, it is very difficult to differentiate them. Thus, to increase the accuracy of classification, the boundary of the urban areas extracted from the 2000 LULC map was used to mask out the pixels of the urban areas within the 2000 and 2009 Landsat images. A modified version of the Chinese Land Resource Classification System was used for classification, including six classes (agricultural land, forest, rangeland, water, barren land and perennial snow or ice). Within this classification system, water and perennial snow or ice is separated into two classes to show the glacial extent of the study area. Over 600 sites covering the six major land cover types were selected as the training sites in the study area. The 2000 LULC map and the shape files of roads and river flow network were used to help identifying the training sites. In addition, a normalized differential vegetation index (NDVI) image calculated in ENVI was also produced to better differentiate between vegetated and barren land areas. Then, the 2000 and 2009 images within the study area were classified using ENVI employing a maximum likelihood classification. Each pixel was assigned to a class that has the highest probability. 

**Table 2:** The Chinese Land Resource Classification System.

Level 1		Level 2	
1	Cropland	11	Paddy
		12	Dry farmland
2	Forestland	21	Forest land
		22	Brush land
3	Grassland	31	High degree overlay grassland
		32	Middle degree overlay grassland
		33	Low degree overlay grassland
4	Water	41	River and channel
		42	Lake
		43	Reservoir
		44	Ice and snow
5	Urban and/or Built-up land	51	City and town
6	Barren land	61	Sand land
		62	Gobi
		63	Saline-alkali-land
		65	Barren land
		66	Rock and gravel land

To evaluate the changes within the agricultural land, but avoid the influence of the wetlands in the middle reach of the study area, we chose one scene satellite image (path 133, row 33) from both 2000 and 2009 data with little or no wetland distribution. We then masked out the agricultural land using the classification results, and then further classified it into three groups: corn, wheat and rice areas, also using a maximum likelihood classifier as described above. The selected image (path 133, row 33) was also ideal for distinguishing these three crops in the area, as among the multiple dates of the images acquired only this scene from August met the requirement for crop chronology analysis. In the study area, spring wheat is normally harvested by July, and corn is harvested in September. Use of August satellite images for classification is relatively more effective for differentiating the wheat from the corn planting area in the study region.

Accuracy assessment was conducted to evaluate the classification results. Sample points were selected using a stratified random method. The sample size was selected from a sample of 10% of the pixels proportional to the total number of pixels in each of the sample classes. The LULC map of 2000 from the Chinese Academy of Sciences was used as a ground truth reference for assessing the accuracy of the image classification for 2000. For the 2009 image classification maps of both the study area and the agricultural land area within the subset area, the 2008 air photo from the Chinese Academy of Sciences was used as a reference as this is the only high resolution data available. Contingency tables were derived to indicate the producer’s accuracy, the user’s accuracy, overall accuracy, and the Kappa coefficient. Each row of the table represents the truth in a predicted class, while each column represents the results of image-derived classification.

## 3. Results

### 3.1. Classified LULC in the Middle Reach of the HRB

**Figure 2:**
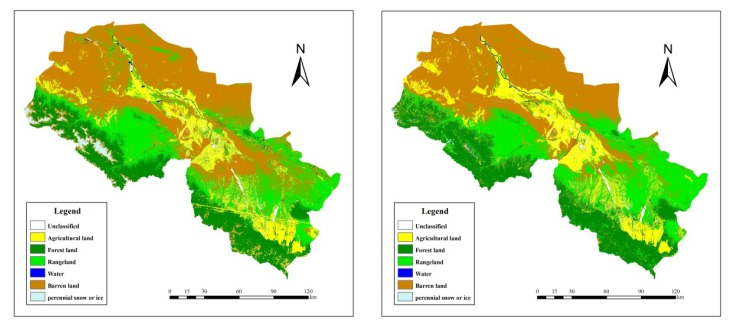
The LULC maps of the middle reach of the HRB for 2000 (left) and 2009 (right).

Figure 2 shows both 2000 and 2009 LULC classification maps. The contingency tables (confusion matrices) (Table 3 and Table 4) indicate the degree of misclassification among all the classes derived from the images. Each confusion matrix contains the overall accuracy percentage and the Kappa coefficient, and the classification accuracy is assessed through evaluating the overall classification accuracy and Kappa Statistics [22]. 

The overall classification accuracies are 86.53% and 85.80% in 2000 and 2009, respectively (Table 3 and Table 4). The Kappa statistics are 0.77 for both the 2000 and 2009 results. In 2000 and 2009 LULC classification confusion matrices, the accuracy rates of rangeland are relatively low, meaning actual rangelands were misclassified to other types, such as agricultural and barren land due to the similar spectral reflectance values of the rangeland, agricultural region and barren land within the particular region. 

According to the 2000 LULC map, 43.25% of the middle reach of the HRB (total 27,955 km^2^) was dominated by barren land (12,089.43 km^2^). 22.23% of the land was forest (6,213.26 km^2^) and 18.39% was rangeland (5,142.19 km^2^). Agricultural land (3,685.30 km^2^) constituted 13.18% of the study area. The remaining 1.67% of the middle reach of the HRB (467.95 km^2^) was covered by water, ice and snow (Table 5).

**Table 3:** Contingency table of derived 2000 LULC map.

Land Cover Type	Agriculture	Forest	Rangeland	Water	Barren	Ice and Snow	Total	User’s Accuracy
Agriculture	**80.25**	3.66	1.87	0	0.39	0	5.16	78.34
Forest	2.45	**89.68**	21.03	2.25	0.05	0	17.31	77.04
Rangeland	8.93	1.43	**71.89**	0	9.19	0	19.26	67.75
Water	0	0.02	0	**97.71**	0	0	0.08	95.96
Barren	8.37	4.08	5.21	0.04	**90.34**	0.09	56.54	96.51
Ice and Snow	0	1.10	0	0	0	**99.91**	1.63	89.99
Total	100	100	100	100	100	100	**100**	
Producer’s Accuracy	80.25	89.68	71.89	97.71	90.34	99.91		
Overall Accuracy = **86.53%**; Kappa Coefficient = 0.77.								

**Table 4:** Contingency table of derived 2009 LULC map.

Land Cover Type	Agriculture	Forest	Rangeland	Water	Barren	Ice and Snow	Total	User’s Accuracy
Agriculture	**77.25**	2.62	2.25	0.06	0.42	0	5.54	79.72
Forest	6.32	**94.62**	11.64	0.31	0.11	0	20.80	88.86
Rangeland	15.43	2.52	**80.20**	0	14.23	0	22.72	57.40
Water	0	0.01	0	**99.46**	0	0.04	0.06	96.61
Barren	1	0.18	5.91	0.17	**85.21**	0.00	50.78	97.92
Ice and Snow	0	0	0	0	0	**99.96**	0.07	99.75
Total	100	100	100	100	100	100	**100**	
Producer’s Accuracy	77.25	94.62	80.20	99.46	85.21	99.96		
Overall Accuracy = **85.80%**; Kappa Coefficient = 0.77.								

**Table 5:** 2000 and 2009 classified LULC types, area, percentage, and changes.

Land Cover Type	2000		2009		Changes
	Area (km^2^)	Percentage (%)	Area (km^2^)	Percentage (%)	of Area
Agriculture	3,685.30	13.18	4,431.12	15.85	745.82
Forest	6,213.26	22.23	6,850.19	24.50	636.93
Rangeland	5,142.19	18.39	5,993.69	21.44	851.5
Water	31.78	0.11	77.63	0.28	45.85
Barren	12,089.43	43.25	10,195.99	36.47	−1,893.44
Ice and Snow	436.17	1.56	50.42	0.18	−385.75

Similarly, in 2009, barren land in the area decreased to 10,195.99 km^2^, but was still the major land use type in the study area. The areas of forest (6,850.19 km^2^), rangeland (5,993.69 km^2^) and agricultural land (4,431.12 km^2^) have minor increases, which constituted 24.50%, 21.44% and 15.85% of the middle reach of the HRB, respectively. About 0.46% of the study area (128.05 km^2^) was covered by water, ice and snow (Table 5). 

### 3.2. Agricultural Types in the Selected Subset of the Study Area

**Figure 3:**
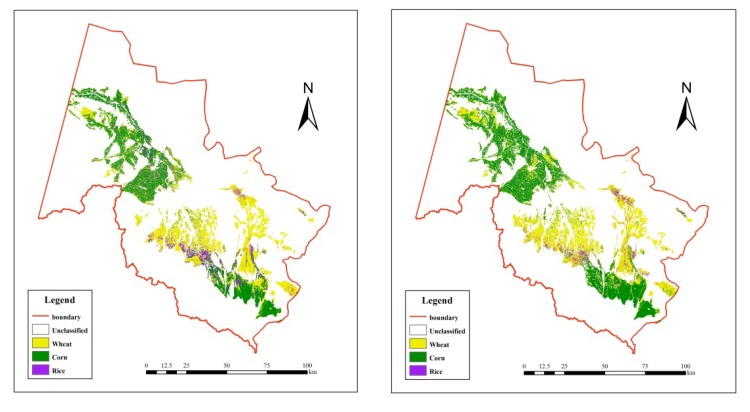
The agricultural types within the selected subset in August, 2000 (left) and 2009 (right).

Similar classification procedures were conducted in the selected subset area, using the selected images (Path 133, Row 33) in August 2000 and August 2009, respectively. The agricultural land was further classified into rice, wheat and corn, as shown in Figure 3. The overall accuracies of the classification are 88.60% and 89.92% in 2000 and 2009, respectively. The Kappa coefficient is 0.76 and 0.79, respectively (Table 6 and Table 7).

The areas of each crop types are calculated to identify the changes of each type. In 2000 the areas of wheat, corn and rice within the selected subset are 1,606.33 km^2^_,_ 1,352.29 km^2^ and 296.81 km^2^, respectively. While in 2009, the area of wheat plant was 1,720.11 km^2^, corn area was 1,735.53 km^2^, and rice area was 150.64 km^2^.

**Table 6:** Contingency table of 2000 LULC of agricultural land from a single Landsat scene (Path 133 Row 33).

Crop	Wheat	Corn	Rice	Total	User’s Accuracy
Wheat	**97.17**	10.71	18.64	35.47	78.32
Corn	1.08	**85.19**	0.06	60.67	99.49
Rice	1.57	3.17	**81.30**	3.15	14.50
Total	100.00	100.00	100.00	**100.00**	
Producer’s Accuracy	97.17	85.19	81.30		
Overall Accuracy = 88.60%; Kappa Coefficient = 0.76.					

**Table 7:** Contingency table of 2009 LULC of agricultural land from a single Landsat scene (Path 133 Row 33).

Crop	Wheat	Corn	Rice	Total	User’s Accuracy
Wheat	**92.44**	10.91	25.52	39.84	82.21
Corn	2.75	**88.61**	0.00	57.92	98.32
Rice	4.61	0.41	**74.48**	2.12	10.66
Total	100.00	100.00	100.00	**100.00**	
Producer’s Accuracy	92.44	88.61	74.48		
Overall Accuracy = 89.92%; Kappa Coefficient = 0.79.					

## 4. Discussion

**Figure 4:**
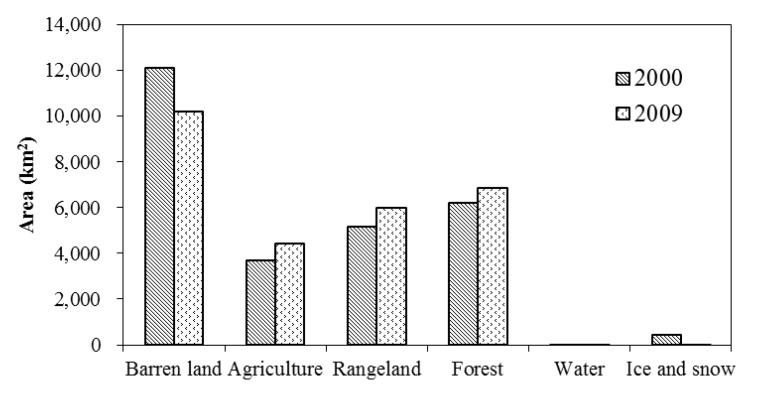
Changes in LULC classes from 2000 to 2009.

**Figure 5:**
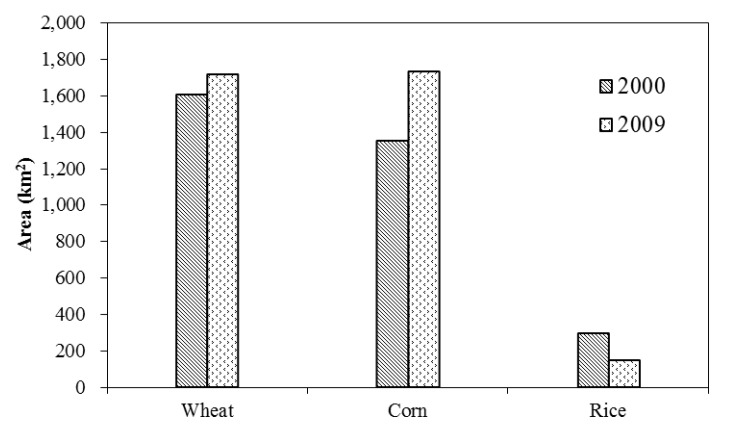
Changes of wheat, corn, and rice planting areas between 2000 and 2009.

Comparing the two classification maps as showed in Figure 2, LULC change is significant: the barren land largely decreased (decreased by 1,893 km^2^), especially in the middle part of the study area. Simultaneously, the areas of agricultural land, range land and forest had expanded by about 745 km^2^, 850 km^2^ and 636 km^2^ respectively (Figure 4). The increase of agricultural land mainly happened in the region previously to be barren land (Figure 2). More specifically, between 2000 and 2009 within the selected subset of the study area, rice planting area had declined by about 146 km^2^, while wheat and corn planting area both increased (Figure 5).

This change would enable the middle reach to deliver more water to the lower reach because wheat and corn uses much less water [19]. In a similar study, Qi and Luo [12] estimated the LULC changes in the HRB from 1987 to 2002 using Landsat data, and reported that cropland and urban area increased by 174.9, and 64.6 km^2^, and grassland decreased by 210.3 km^2^ respectively, in the middle reach of the HRB during the period. Gu *et al.* [23] used time-series NDVI data and geographical ancillary data to classify the LULC in the HRB and, with an overall classification accuracy of 72% and the Kappa index of 0.68. Our findings are similar to those by Qi and Luo [12] and Gu *et al.* [23]. While lack of high resolution data and field investigations contributed to classification inaccuracies, nevertheless, the changes at least partially reflect the changing crop pattern in the middle reach of the HRB.

**Figure 6:**
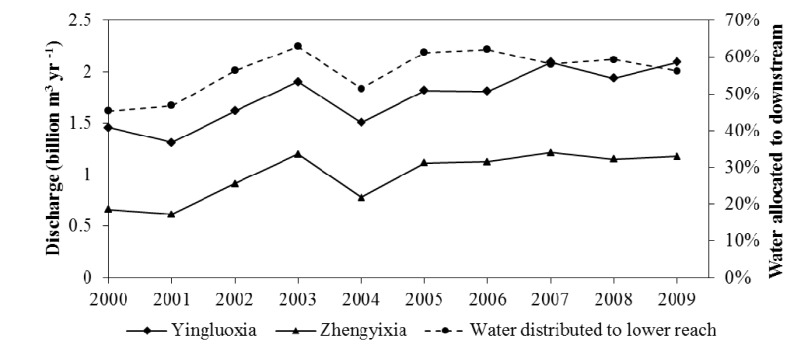
Discharge of the Heihe River at the Yingluoxia and Zhengyixia gauge stations and Percentage of the total flow distributed to the lower reach of the Heihe River Basin.

**Figure 7:**
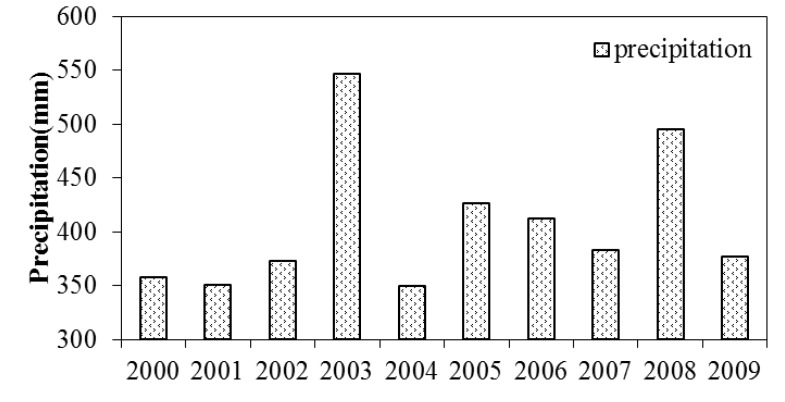
Annual precipitation of the Qilian station from 2000 to 2009.

The impacts of adjusted crop patterns on water allocation can be assessed from the percentage of water allocated to the lower reach calculated from the streamflow data. The Yingluoxia and Zhengyixia gauge stations are located at the outlet of the upper and middle reach of the HRB, respectively. The water of Heihe River goes through the Yingluoxia gauge station to the middle reach and passes the Zhengyixia gauge station to the lower reach (Figure 1). Figure 6 displays the discharges of the two gauge stations from 2000 to 2009 and also the percentage of water distributed to the lower reach through the Zhengyixia gauge station over the incoming water from the Yingluoxia gauge station. After 2000, the streamflow at the Zhengyixia gauge station increased except 2004, when the flow from the Yingluoxia gauge station also declined significantly. The relationship between the precipitation of the Qilian station from 2000 to 2009 (Figure 7) and streamflow through the Yingluoxia gauge station is also evaluated. 

There is significant correlation (*r* = 0.767, *p* = 0.016) between the annual precipitation and the streamflow, large amount of annual precipitation leads to higher streamflow, but the increased percentage of the discharge at the Zhengyixia gauge station to the incoming flow at the Yingluoxia gauge station stays at a relative higher rate when the precipitation seems decreasing since 2005. This appears to indicate that the changing agricultural crop patterns have largely affected the water allocation leading to the delivery of the required discharge to the lower reach. Our findings are similar to those of Wu *et al.* [24] and Wang *et al.* [25]. Wu and his colleagues [24] analyzed the long-term trend of the hydrometeorological variables including air temperature and precipitation (for the period of 1959–2009) and discharges (for the period of 1978–2007) in the Upper Reach of the HRB using the nonparametric Mann-Kendall trend test. They report that the air temperature had increased significantly (α = 0.05) for the study period [24]. However, both the precipitation and discharges didn’t display a statistically significant increase for the same period [24]. Wang and his colleagues [25] who report that land use changes in the middle reach of the HRB significantly impacted the spring and winter runoff and base flow of Heihe River, and adjusting the land use structure would lead to increasing discharge to the lower reach by approximately 9%, helping the restoration and reconstruction of the ecosystem in the downstream area.

## 5. Conclusions

This study conducted a comparative analysis of the changes in LULC for the period from 2000 to 2009 within the middle reach of the HRB, Northwest China using Landsat 5/Landsat 7 satellite data. The results show that, from 2000 to 2009, agricultural land, forest area, rangeland, and water area were increased, while barren land area was decreased. Within the cropland, the rice planting area had decreased, while wheat and corn planting areas had increased. These changes reduced the water consumption in the middle reach of the river and ensured the discharge of the mandated flow to the lower reach, rehabilitating the East Juyan Lake and West Juyan Lake ecosystems downstream.

This study uses a qualitative approach to assess changing crop patterns and associated impacts on water resources, which is simple, fast, and particularly useful in areas such as the HRB where land use/cover data with adequate spatial and temporal resolution are lacking. However, results from such assessment need to be further verified with *in situ* data before being used in support of land use planning and water resources management.
